# The heart in systemic lupus erythematosus – A comprehensive approach by cardiovascular magnetic resonance tomography

**DOI:** 10.1371/journal.pone.0202105

**Published:** 2018-10-01

**Authors:** Thilo Burkard, Marten Trendelenburg, Thomas Daikeler, Christoph Hess, Jens Bremerich, Philip Haaf, Peter Buser, Michael J. Zellweger

**Affiliations:** 1 Department of Cardiology, University Hospital Basel, Basel, Switzerland; 2 Medical Outpatient Department, University Hospital Basel, Basel, Switzerland; 3 University of Basel, Basel, Switzerland; 4 Division of Internal Medicine, University Hospital Basel, Basel, Switzerland; 5 Department of Rheumatology, University Hospital Basel, Basel, Switzerland; 6 Department of Radiology, University Hospital Basel, Basel, Switzerland; Keio University, JAPAN

## Abstract

**Background:**

In systemic lupus erythematosus (SLE), cardiac manifestations, e.g. coronary artery disease (CAD) and myocarditis are leading causes of morbidity and mortality. The prevalence of subclinical heart disease in SLE is unknown. We studied whether a comprehensive cardiovascular magnetic resonance (CMR) protocol may be useful for early diagnosis of heart disease in SLE patients without known CAD.

**Methods:**

In this prospective, observational, cross-sectional study CMR including cine, late gadolinium enhancement (LGE) and stress perfusion sequences, ECG, and blood sampling were performed in 30 consecutive SLE patients without known CAD. All patients fulfilled at least 4/11 American College of Rheumatology (ACR) Criteria for the classification of SLE.

**Results:**

30 patients (83% female) were enrolled, mean age was 45±14 years and mean SLE disease duration was 10±8 years. 80% had low to moderate disease activity. All had a low SLE damage index. CMR was abnormal in 13/30 (43%), showing LGE in 9/13, stress perfusion deficits in 5/13 and pericardial effusion (PE) in 7/13. Patients with non-ischemic LGE had more often microalbuminuria while patients with stress perfusion deficits a history of hypertension, renal disorder as ACR criterion, repolarisation abnormalities on ECG and larger LV enddiastolic volume index. There was no correlation between clinical symptoms and CMR results.

**Conclusion:**

Our study shows that cardiac involvement as observed by CMR is frequent in SLE and not necessarily associated with typical symptoms. CMR may thus help to detect subclinical cardiac involvement, which could lead to earlier treatment. Additionally we identify possible risk factors associated with cardiac involvement.

## Introduction

Systemic lupus erythematosus (SLE) is a systemic, autoimmune disease which can affect virtually all organs[1]. Cardiac involvement is a leading cause of morbidity and mortality[2]. All anatomic structures of the heart may be affected in SLE patients. Pericarditis is part of the diagnostic criteria of the American College of Rheumatology (ACR), accelerated atherosclerosis leading to premature coronary artery disease (CAD), myocardial involvement and valvular heart disease with abacterial endocarditis have been described[3–5]. Thereof CAD and myocardial involvement are of particular interest since both conditions may comprise an adverse prognosis. For example, in young women with SLE a 50-fold increase of myocardial infarction (MI) has been described with MI being the cause of death in up to 30% of SLE patients[6]. In fact, cardiovascular events are major contributors to premature death in patients with SLE and in spite of a declining all-cause mortality over the past decades, cardiovascular mortality remains unchanged[7]. In autopsy studies evidence of myocardial involvement is found in up to 40% but is clinically diagnosed in only 10% of patients [8–10]. This diagnostic gap may not be closed by echocardiography since only 20% of asymptomatic patients showed left ventricular (LV) abnormalities, such as like LV dilatation, LV hypertrophy or LV dysfunction[11].

Therefore we studied the diagnostic yield of a comprehensive, routine cardiac magnetic resonance (CMR) examination, including adenosine-stress perfusion and late gadolinium enhancement (LGE) in SLE patients without history of coronary artery disease (CAD).

## Materials and methods

### Study population

In this prospective, observational, cross-sectional study we included a total of 30 consecutive patients with the diagnosis of SLE as defined by the American College of Rheumatology (ACR) Classification Criteria[3, 4]. Exclusion criteria were: known CAD, age < 18 years, impaired renal function (estimated glomerular filtration rate less than 30 ml/min, by the Modification of Diet in Renal Disease Study Group (MDRD) formula[12]), non-MR-safe devices or ferromagnetic implants, metallic foreign bodies in the eye, known allergies against CMR contrast media or adenosine, presence of chronic atrial fibrillation, 2^nd^ or 3^rd^ degree atrioventricular block, trifascicular block, asthma and severe chronic obstructive pulmonary disease. Patients were recruited consecutively between 2010 and 2014 at the University Hospital Basel in collaboration with the Divisions of Rheumatology and Immunology and the Swiss SLE Cohort Study (SSCS)[13]. The study protocol complies with the Declaration of Helsinki and was approved by the Ethics Commission of the Cantons of Basel (EKBB-Nr. 45/10). Prior to study inclusion informed consent was obtained.

Case history, SLE disease duration, disease activity as well as SLE disease related organ damage and recent medication were provided by the treating physician after a routine outpatient visit or during inpatient hospitalisation. For the evaluation of SLE disease activity the European Consensus Lupus Activity Measurement (ECLAM) score (ranging from 0 indicating no activity to 16.5 as maximum) and the Physician’s Assessment of Disease Activity (ranging from 0 = inactive, 1 = moderately active, 2 = active to 3 = very active) were used[14]. For SLE disease related organ damage, the Systemic Lupus Erythematosus International Collaborating Clinics/American College of Rheumatology (SLICC/ACR) damage index (ranging from 0 indicating no damage to 47 as maximum) was calculated[15].

At the day of the CMR, a cardiologist obtained a detailed cardiovascular history and a clinical exam was performed. For the classification of chest symptoms we chose the definition of angina pectoris by the European Society of Cardiology [16]. Accordingly, typical angina pectoris meets all three of the following characteristics: substernal chest discomfort of characteristic quality and duration; provoked by exertion or emotional stress; relieved by rest and/or nitrates within minutes. Atypical angina meets two of these characteristics. Non-anginal chest pain lacks or meets only one or none of the characteristics.

Additionally an ECG and laboratory investigations were done (including high-sensitivity troponin and brain natriuretic peptide). ECGs were interpreted by an experienced cardiologist (TB, MZ). Repolarisation abnormalities were defined as ST-segment elevation, depression or discordant T-wave inversions.

### CMR data acquisition and analysis

Patients underwent routine CMR including perfusion studies at stress and rest as well as LGE sequences in a mean time frame of 23 days (interquartile range 25–75 [8,50]) before or after the index visit with their treating physician.

Routine ECG-gated CMR studies were performed in the supine position on a 1.5 Tesla magnet (Magnetom Avanto, Siemens, Germany) equipped with phased array body coils for signal reception using commercially available cardiac software. Heart rate and ECG were continuously monitored, blood pressure every two minutes. The stress test to evaluate ischemia was performed by infusion of 140 μg/kg/min adenosine. After 3 minutes of adenosine, acquisition of breath-hold saturation recovery gradient recalled echo (SR-GRE) images and injection of contrast media were started simultaneously. Adenosine infusion was stopped upon completion of stress perfusion MR. Rest perfusion images were acquired without adenosine but otherwise with identical contrast material settings and sequence parameters as mentioned before for stress. Cine images for functional assessment were acquired with ECG gated 2D steady state free precession (cineTrueFisp) in a stack of short axis, 2-chamber and 4-chamber views. For late enhancement images inversion recovery turbo fast low angle shot (IR-tFLASH) images were acquired ~15 min after a cumulative dose of 0.2 mmol/kg GdDOTA in the short axis, 2-chamber and 4-chamber views. The inversion time was optimized in order to obtain null signal from normal myocardium. CMR analyses were performed off-line by experienced readers. Regional wall motion, perfusion and late gadolinium enhancement were interpreted by consensus reading of an experienced cardiologist (MZ, PB) and an experienced radiologist (JB).

Abnormal CMR was defined as presence of LGE (ischemic or non-ischemic pattern), signs of stress-perfusion deficit indicating ischemia or pericardial effusion (PE). Pericardial effusion was visually categorized by consensus reading to (0 = no, 1 = mild, 2 = moderate, 3 = large). As LV and right ventricular (RV) functional parameters, LV ejection fraction (LVEF) and RV ejection fraction (RVEF) were calculated quantitatively. Normal LVEF was defined as >54%, normal RVEF as >45%. LV mass, LV enddiastolic volume, LV stroke volume as well as RV enddiastolic volume were calculated and indexed on body surface area.

### Statistical analysis

Categoric variables are expressed as counts (percentage). Continuous variables are expressed as mean ± standard deviation (SD) if normally distributed, or as median and interquartile range if not normally distributed. Comparisons were performed using Student’s t-test, Mann—Whitney U-test or Pearson chi-square test, as appropriate. A p-value of <0.05 was considered statistically significant, significance levels are two-tailed, and confidence intervals (CI) are 95%-CI. All calculations were done with the use of the SPSS statistical package version 22.0 (SPSS Inc., Chicago, IL, USA).

## Results

In all we screened 51 patients for participation in the study. 20 patients were excluded prior to CMR (4 due to known coronary artery disease, another 4 due to renal insufficiency, 2 due to claustrophobia, 9 refused informed consent and one died before baseline visit). One patient discontinued CMR after localizer sequences due to claustrophobia and was thus excluded from the analysis. Two patients did not receive adenosine stress testing since they had normal coronary artery status in a recent coronary angiography and refused additional stress testing, resulting in 28 patients with adenosine stress testing. Baseline characteristics of the final cohort of 30 patients are shown in Table 1, ECG parameters and laboratory results in Table 2 and SLE specific treatment at time of CMR is listed in Table 3.

**Table 1:** Baseline characteristics of the cohort.

**Female sex**	83%
**Participants of the Swiss SLE Cohort Study**	83%
**Age in years**	45 ± 14
**Body mass index in kg/m^2^**	23.8 ± 6
**History:**	
• **Dyspnoea**	36.7%
• **Angina pectoris**	0%
• **Atypical angina pectoris**	33.3%
• **Non-anginal chest pain**	26.7%
**Cardiovascular risk factors:**	
• **Dyslipidemia**	50%
• **Arterial Hypertension**	33.3%
• **Family history of cardiovascular disease**	13.3%
• **Active or former smoking**	33%
• **Diabetes mellitus**	3.3%
**Lupus parameters:**	
• **Disease duration in years**	10.1 ± 8.1
• **ECLAM score**	1.5 [0, 2.5]
• **Physician’s global assessment of disease activity**	0 [0, 1]
• **SLICC/ACR damage index**	0 [0, 1]
• **ACR Criteria for the classification of SLE**	5 [4, 6.25]
• **Photosensitivity**	53%
• **Malar rash**	50%
• **Discoid rash**	10%
• **Oral ulcers**	17%
• **Arthritis**	73%
• **Pleuritis**	23%
• **Perikarditis**	23%
• **Renal disorder**	30%
• **Hematological disorder**	50%
• **Seizures**	6.7%
• **Psychosis**	17%
• **Antinuclear-Antibody**	90%
• **Anti-DNA-Antibody**	83%
• **Anti-Sm nuclear antigen-Antibody**	17%
• **Anti-Phospholipid-Antibody**	23%
**Physical examination:**	
• **Systolic blood pressure in mmHg**	118 ± 15
• **Diastolic blood pressure in mmHg**	72 ± 13
• **Heart rate per minute**	72 ± 10
• **Systolic murmur on cardiac auscultation**	10%
• **Diastolic murmur on cardiac auscultation**	3.3%
• **Rales on pulmonary auscultation**	3.3%
• **3^rd^ heart sound**	0%
• **Peripheral oedema**	10%
• **Positive hepatojugular reflux**	0%
• **Congestion of jugular veins**	0%
• **Diminished peripheral pulses**	3.3%

Data are given as percentages; mean±SD; median [IQR] as appropriate. ECLAM—European Concensus Lupus Activity Measurement; SLICC/ACR—Systemic Lupus International Collaborative Clinics/American College Rheumatology

**Table 2:** ECG parameters and laboratory results of the SLE patients at baseline.

**Electrocardiogram**	
• **Sinus rhythm**	96.7%
• **Atrial flutter**	3.3%
• **Atrioventricular block**	0%
• **PQ time in ms**	154±19
• **QRS width in ms**	87±15
• **QTc in ms**	420±26
• **Incomplete right bundle branch block**	6.7%
• **Left bundle branch block**	3.3%
• **Q-waves in II, III, aVF**	0%
• **Repolarisation abnormalities**	20%
**Laboratory results**	
• **Brain natriuretic peptide in ng/l (normal value <50.3)**	32 [21, 58]
• **High sensitivity Troponin in μg/l (normal value <0.014)**	<0.003 [<0.003; 0.055]
• **Microalbuminuria**	22%

Data are given as percentages; mean±SD; median [IQR] as appropriate.

**Table 3:** SLE specific treatment at the time of the CMR.

**Glucocorticoids**	56.7%
**Hydroxychloroquin**	76.7%
**Chloroquin**	3.3%
**Rituximab**	(3.3%)
**Azathioprine**	(6.7%)
**Mycophenolate mofetil**	(23.3%)

Data are given as percentages. SLE–Systemic lupus erythematosus; CMR—cardiovascular magnetic resonance.

### CMR findings

**Fig 1:**
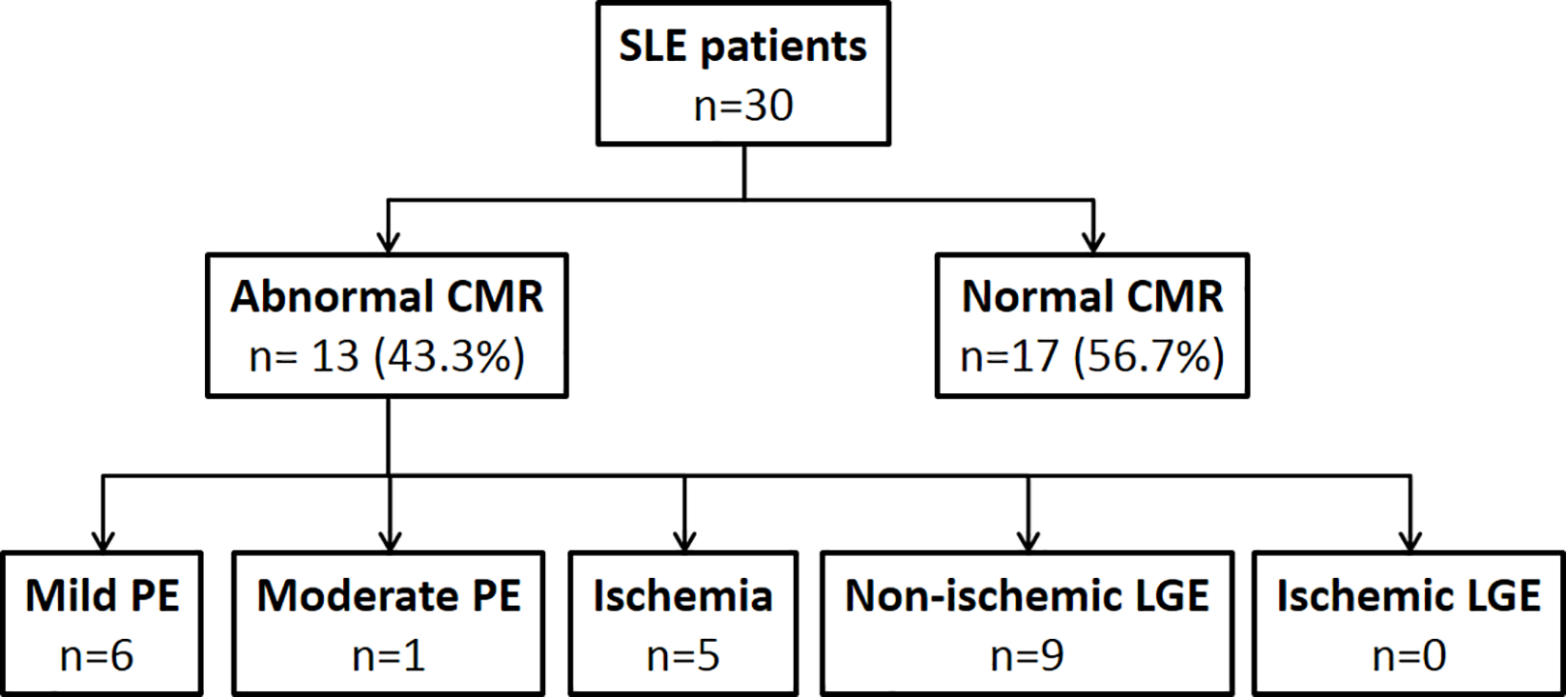
Structural and functional abnormal CMR findings. PE–Pericardial effusion; LGE–late gadolinium enhancement; Ischemia indicating stress-perfusion deficit.

CMR was interpreted as abnormal in a total of 13 patients (43.3%). An overview of the observed abnormalities is given in Fig 1.

In functional imaging all except one patient had normal LVEF (mean LVEF 68±6%). Mean left ventricular mass index (LVMi) was 51±14 g/m^2^, mean left ventricular enddiastolic volume index (LVEDVi) 77±15 ml/m^2^. The LVEF of the above-mentioned patient was slightly reduced to 53% and CMR showed additionally LGE of a non-coronary pattern, regional wall motion abnormalities and a stress perfusion deficit. RVEF was normal in all patients (mean RVEF 61±8%).

**Fig 2:**
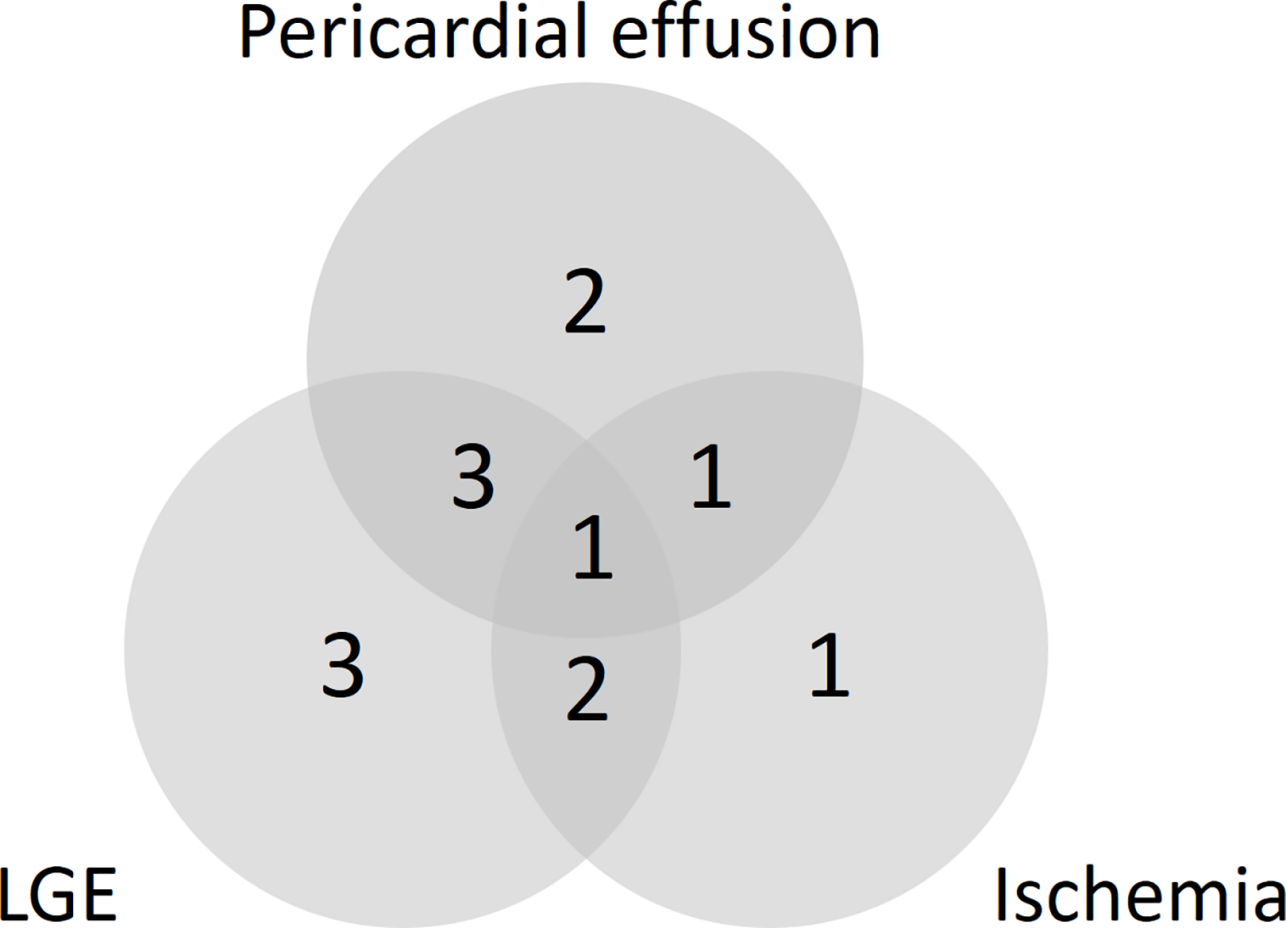
Frequency and combinations of structural or functional abnormalities in patients with abnormal CMR. LGE–late gadolinium enhancement; Ischemia indicating stress-perfusion deficit.

Regarding the 13 patients with abnormal CMR, there were different combinations of CMR findings observed with 6/13 patients showing one structural or functional abnormality, 6/13 patients showing combination of two abnormalities and 1/13 patients showing three abnormalities. Frequency of the observed structural abnormalities and combinations are shown in Fig 2.

**Fig 3:**
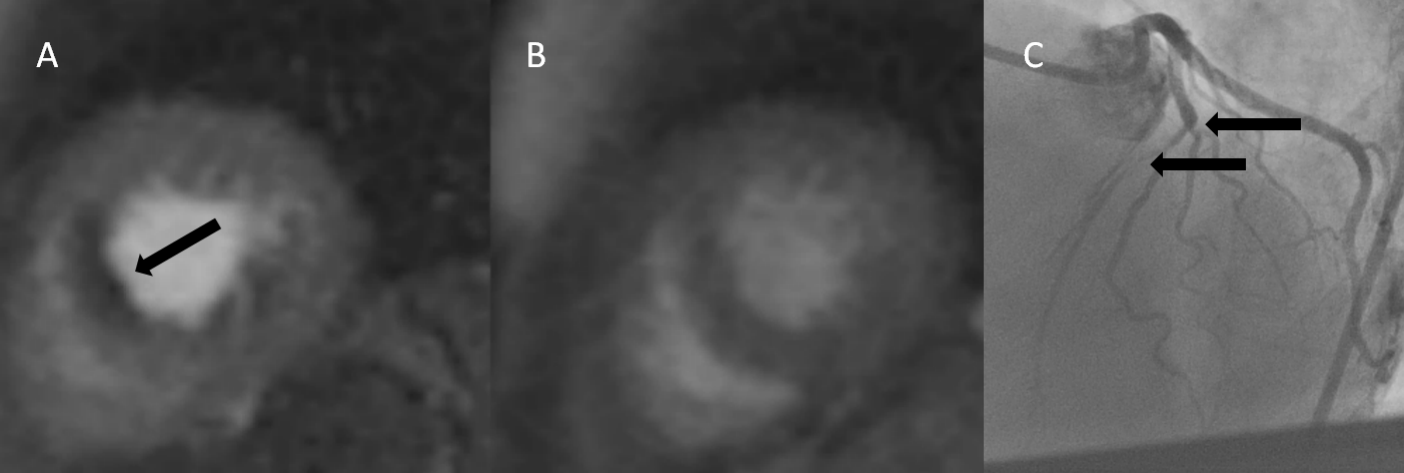
CMR and coronary angiography in one case with ischemia and coronary artery disease. A: Short axis stress-perfusion image showing anteroseptal perfusion deficit (arrow). B: Corresponding rest-perfusion. C: Coronary angiography with significant stenoses of the left anterior descending artery including its bifurcation to the diagonal branches (arrows).

In one patient with stress-perfusion deficit coronary angiography was performed showing significant coronary two-vessel disease (Fig 3).

### Comparison of patients with versus without late-gadolinium enhancement and potential clinical predictors

Patients with LGE had microalbuminuria more frequently than patients without LGE (33,3% vs. 9,5%), p = 0.048). There were no other significant differences with regard to SLE manifestations, the above-mentioned baseline characteristics or functional CMR results. Details are provided in Table 4.

**Table 4:** Comparison between patients without versus with the presence of LGE on CMR.

	No LGE (n = 21)	LGE (n = 9)	p-value
**Female sex**	85.7%	77.8%	0.223
**Age in years**	43±14	48±15	0.449
**Cardiovascular risk factors**			
• **Dyslipidemia**	52%	44%	0.5
• **Hypertension**	29%	44%	0.331
• **Family history of cardiovascular disease**	14%	11%	0.655
• **Active or history of smoking**	28%	44.4%	0.398
• **Diabetes**	5%	0%	0.7
**Lupus parameters**			
• **Disease duration in years**	9.1±6.7	12.6±10.5	0.476
**Laboratory results**			
• **Brain natriuretic peptide in ng/l**	32 [18;51]	43 [21;205]	0.158
• **High sensitivity Troponin in μg/l**	<0.003 [<0.003;0.0045]	0.0034 [<0.003;0.016]	0.351
• **Microalbuminuria**	9.5%	33.3%	0.048

Data are given as percentages; mean±SD; median [IQR] as appropriate. LGE–late gadolinium enhancement

### Comparison of patients with versus without stress-perfusion deficit and potential clinical predictors

Stress-perfusion deficits were found in five of 28 patients with adenosine-stress perfusion imaging. Comparing patients with versus without stress-perfusion deficit, there were significant differences regarding hypertension as cardiovascular risk factors, presence of renal disorder as ACR criterion for the classification of SLE, repolarisation abnormalities on ECG, brain-natriuretic peptide and functional left ventricular CMR parameters (see Table 5). There were no significant differences regarding SLE disease duration, SLE disease activity or SLE related organ damage.

**Table 5:** Comparison between patients without versus with stress-perfusion deficit on CMR.

	No stress-perfusion deficit (n = 23)	Stress-perfusion deficit (n = 5)	p-value
**Female sex**	87%	80%	0.905
**Age in years**	43±13	47±19	0.771
**History**			
• **Atypical Angina pectoris**	35%	20%	0.521
• **Non-anginal chest pain**	30%	20%	0.640
**Cardiovascular risk factors**			
• **Dyslipidemia**	48%	60%	0.622
• **Hypertension**	22%	80%	0.011
• **Family history of cardiovascular disease**	13%	0%	0.393
• **Active or former smoking**	16%	60%	0.141
• **Diabetes**	4.3%	0	0.635
**ACR Criteria for the classification of SLE**			
• **Photosensitivity**	61%	20%	0.097
• **Malar rash**	44%	80%	0.139
• **Discoid rash**	9%	0%	0.494
• **Oral ulcers**	13%	20%	0.687
• **Arthritis**	74%	80%	0.776
• **Pleuritis**	22%	20%	0.393
• **Pericarditis**	22%	20%	0.393
• **Renal disorder**	22%	80%	0.011
• **Hematological disorder**	48%	60%	0.622
• **Seizures**	9%	0%	0.494
• **Psychosis**	22%	0%	0.250
• **Antinuclear-AB**	91%	80%	0.459
• **Anti-DNA-AB**	87%	60%	0.154
• **Anti-Sm nuclear antigen-AB**	17%	20%	0.890
• **Anti-Phospholipid-AB**	22%	40%	0.393
**Electrocardiogram**			
• **Repolarisation abnormalities**	4%	60%	0.001
**Laboratory results**			
• **Brain natriuretic peptide in ng/l**	29.3 [15.8;47.8]	68.6 [28.2;183.7]	0.033
• **High sensitivity Troponin in μg/l**	<0.003 [<0.003;0.0032]	0.006 [<0.003;0.7]	0.373
• **Microalbuminuria**	13%	20%	0.294
**CMR parameters**			
• **Pericardial effusion**	23%	20%	0.393
• **LVEF in %**	68±5	63±5	0.102
• **LV enddiastolic volume index in ml/m^2^**	73±12	91±17	0.027

Data are given as percentages; mean±SD; median [IQR] as appropriate. AB–Antibody; ACR–American College Rheumatology; LVEF–left ventricular ejection fraction

## Discussion

To our knowledge, this is the first study using a comprehensive, routine cardiac magnetic resonance (CMR) examination including functional assessment, adenosine stress-perfusion imaging and late gadolinium enhancement to evaluate cardiac involvement in oligosymptomatic SLE patients without known coronary artery disease.

The current study showed, that in more than 40% of patients CMR yielded abnormal findings including pericardial effusion, LGE of a non-ischemic pattern or stress-perfusion deficits. Most frequently we observed non-ischemic LGE and PE but in 20% of patients we found adenosine-stress perfusion deficits as sign of coronary artery disease. In more than half of the SLE patients with abnormal CMR we found a combination of two or more structural or functional abnormalities.

Previous studies reporting the use of CMR in SLE patients focused on either myocardial tissue characterisation or on myocardial perfusion, but there was a lack of data on the combination of both techniques in the same patient cohort.

### Myocardial tissue characterisation

In the first CMR case-series and studies in SLE patients, myocardial tissue characterisation was usually performed by rather investigational and non-fully standardised techniques such as earlier forms of T1- or T2-mapping. In these studies myocardial signal alterations were found in subsets of SLE patients and were inconsistently related to SLE disease activity, but no LGE imaging was used [17–19]. In one study by Mavrogeni et al. e.g. T2 signal was increased in active SLE patients and comparable to values during viral myocarditis but LGE was only present in the minority and less frequent compared to viral myocarditis [20]. Abdel-Aty et al. examined a total of 20 SLE patients and 13 healthy controls [21]. They found a correlation between the SLE disease activity as measured by the ECLAM score and T2 imaging parameters but not functional parameters when comparing patients with active SLE versus inactive SLE vs healthy controls. LGE studies were only performed in 8 of the 20 patients, with 3 of them showing non-ischemic LGE pattern. Improvement of disease activity was associated with an improvement of the T2 parameters but no follow up was performed with regard to LGE. Seneviratne et al. identified LGE in 15 of 41 subjects. Patients with extensive LGE were found to be older than patients with localised or absent LGE. There was no correlation with SLE disease duration and activity and no perfusion studies were done [22]. Mavrogeni et al. investigated 80 SLE patients with atypical cardiac symptoms or signs and normal echocardiography by CMR including LGE but without stress perfusion imaging and found myocardial abnormalities in 27.5% of the patients due to myocarditis, myocardial infarction or vasculitis [23]. In SLE patients with cardiovascular symptoms presence of LGE was even higher [24]. In our cohort LGE was found in 9/30 (30%) of patients and in 9/13 (69%) of patients with abnormal CMR, respectively. In all patients LGE showed a non-ischemic pattern, so we identified no SLE patient with signs of silent myocardial infarction like it was seen in the study by Mavrogeni et al–but this may relate to the fact that one exclusion criterium in our study was known coronary artery disease. As described previously, there were no differences in functional assessment of the LV and RV and no differences in SLE disease activity, SLE duration, ECG parameters and clinical parameters when comparing patients with versus without LGE. The only borderline significant difference was the presence of microalbuminuria, which was observed in more patients with LGE. Comparing our data with previous cohorts, we included mostly inactive or slightly active SLE patients and SLICC/ACR damage index was low–but disease duration of our patients was similar to those investigated previously. Clues that disease duration may matter comes from one study comparing presence of LGE in children with SLE to adults with SLE showing that SLE is even detectable in children with SLE but with a lower frequency compared to adults [25].

### Stress-perfusion imaging

Adenosine-stress perfusion CMR imaging was studied previously in 22 SLE patients with typical or atypical chest pain in the absence of prior obstructive coronary artery disease evaluated by means of coronary computed tomography angiography [26]. Prevalence of 44% abnormal stress myocardial perfusion tests indicating microvascular coronary dysfunction was reported [26]. A second study by Sun et al. compared myocardial nuclear stress perfusion imaging in 33 female patients with SLE and non-specific symptoms like chest discomfort or dyspnoea to 28 cardiovascular asymptomatic SLE patients and 24 age- and sex-matched adults without SLE. Perfusion abnormalities were present in 27/33 non-specific symptomatic SLE patients and in 12/28 asymptomatic SLE patients. None of the 24 age- and sex-matched control patients showed perfusion abnormalities[27]. Especially in patients with SLE or connective tissue disease and secondary peripheral Raynaud’s phenomenon myocardial perfusion seems to be reduced [24, 28]. Our data showed stress-perfusion deficit in 5/30 (17%) patients and 5/13 (38%) patients with structural or functional abnormal CMR respectively. None of patients in our cohort suffered from typical angina pectoris, but 1/3 reported atypical angina pectoris and nearly 1/3 reported non-anginal chest pain—unrelated to CMR results. Comparing patients with versus without stress-perfusion deficits there were no differences with respect to SLE disease duration, disease activity or clinical symptoms. However patients with stress-perfusion deficits had more frequently a history of hypertension, renal disorder as ACR criterion for the classification of SLE, repolarisation abnormalities on ECG and higher BNP values.

On functional CMR analysis LV enddiastolic volume index was larger.

### Clinical implications

LGE identifies increased interstitial volume e.g. caused by myocardial oedema due to acute or chronic inflammation as well as focal fibrosis and scar tissue [29–31]. LGE has a low prevalence in a middle aged population at low- or intermediate risk and was found in approximately 0.7% [32]. For many different diseases like coronary artery disease, non-ischemic dilated cardiomyopathies and diabetes mellitus it is well studied that the presence of LGE was associated with worse cardiovascular prognosis [33–35]. Especially in sarcoidosis presence of LGE was associated with a significantly increased risk of cardiovascular morbidity and mortality with an odds ratio of 3.0 for all-cause mortality [36, 37]. CMR was the most valuable tool for diagnosis and risk prediction of cardiac involvement in a general sarcoidosis population [37]. In SLE, the gap between clinical and autoptic diagnosis of myocardial disease could be narrowed by the use of CMR when comparing 30% of LGE in our cohort with around 40% myocardial involvement observed in autopsy studies[8, 9]. In different connective tissue diseases (CTD) including SLE, LGE was shown to be present early after the initial diagnosis [38]. Considering that CMR including LGE identifies more patients with silent myocardial disease in SLE and other CTD than echocardiography, CMR should be the preferred imaging modality especially when infrastructure allows a broad availability and expertise.Since there were no good clinical parameters to predict myocardial disease in SLE patients, LGE in CMR may provide better risk prediction. This notion is supported by reports from other cardiomyopathies, where increasing extend of LGE is related to poorer outcome. However, outcome data comparing SLE patients with versus without LGE are not available.

Coronary artery disease is a leading cause of morbidity and mortality in SLE patients and there is an unmet need to identify SLE patients with the highest risk of cardiovascular adverse events early in disease course. In our study repolarisation abnormalities, hypertension, renal disorder as ACR criterion and larger LV enddiastolic volumes were more often observed in patients with stress-perfusion deficits. Additionally reduced myocardial perfusion may be related to secondary peripheral Raynaud’s phenomenon a clinical phenomenon not included in our study [28]. Since clinical symptoms were unspecific, these clinical parameters which were easy to obtain may raise suspicion for CAD in SLE patients in the future and may trigger further evaluation of the patients. Attention should especially be drawn to ECG abnormalities. A previous study related ECG abnormalies in terms of Q-waves in the inferior leads to CMR abnormalities representing acute myocarditis, past myocarditis or past myocardial infarction by the use of CMR including LGE but without stress perfusion imaging [39]. In our cohort, no SLE patient had Q waves but we can add the results about repolarisation abnormalities in ECG.

An algorithm for the cardiovascular work-up of SLE patients was recently proposed together with a systematic review by Mavrogeni et al.[40]. In this algorithm, echocardiography remained the cornerstone for non-invasive techniques for the assessment of cardiovascular involvement since it is inexpensive, widely available and can guide further work-up especially in acute clinical situations. CMR was highlighted as preferred imaging modality in SLE patients without cardiovascular symptoms or in oligosymptomatic patients when ECG or echocardiography were abnormal and in not acutely symptomatic patients when additional clinical or laboratory features were present e.g. ECG abnormalities, inflammation. In these situations, CMR could guide further diagnostic evaluation and treatment and could reduce exposure to radiation. The Achilles’ heel of CMR remains the lower availability and the higher costs [40]. Especially in the asymptomatic or oligosymptomatic SLE patient results of the CMR may be used to rule out structural cardiovascular disease or to guide the necessity of further evaluation like coronary angiography in case of relevant ischemia related to the territory of a coronary artery.

Some limitations, however, need to be mentioned. The current study–although being prospective—was an observational, cross-sectional study in a single center with a limited number of patients especially with regard to statistical considerations and calculations. Between group comparisons should be interpreted with caution, but may help to guide further research. Since 83% of our patients were participants of the Swiss SLE Cohort Study, we had a high quality of baseline documentation regarding clinical SLE parameters.

In some patients with stress perfusion deficits, we have no final diagnosis of CAD since coronary angiography or coronary computed tomography was not performed in all patients–but it is known that with a comprehensive CMR approach combining stress-perfusion and LGE, sensitivity and specificity for CAD diagnosis is 89% and 87% respectively [41].

For CMR acquisition we used a comprehensive routine CMR protocol which can be easily applied, but we did not use newer techniques like T1, T2, T2* and extracellular volume mapping which have recently been recommended by a consensus statement by the Society for Cardiovascular Magnetic Resonance and the European Association for Cardiovascular Imaging–especially in the context of the evaluation of suspected myocarditis and myocardial disease [42]. This was due to the fact, that when defining the CMR protocol for the present study in 2010, we decided to rely on easily applicable standard measurements with a broad application in daily clinical practice.

## Conclusions

Cardiac involvement as demonstrated by CMR is frequently observed (43.3%) in SLE patients without typical cardiac symptoms, so there is a need of thorough investigation of cardiovascular disease in this patient group. CMR therefore may help to detect subclinical cardiac involvement particularly in patients with repolarisation abnormalities on ECG, hypertension or renal disorder and should be preferred as first line non-invasive cardiac imaging modality when available. The prognostic impact of LGE and stress-perfusion deficits on CMR remains to be determined. Further studies should additionally implement newer CMR sequences and cardiac CT in SLE with stress perfusion abnormalities that are compatible with coronary artery disease.
